# Tobacco Smoking in HIV-Infected versus General Population in France: Heterogeneity across the Various Groups of People Living with HIV

**DOI:** 10.1371/journal.pone.0107451

**Published:** 2014-09-09

**Authors:** Laure Tron, France Lert, Bruno Spire, Rosemary Dray-Spira, the ANRS-Vespa2 study group

**Affiliations:** 1 INSERM, UMR_S 1136, Pierre Louis Institute of Epidemiology and Public Health, Department of social epidemiology, Paris, France; 2 Sorbonne Universités, UPMC Univ Paris 06, UMR_S 1136, Pierre Louis Institute of Epidemiology and Public Health, Department of social epidemiology, Paris, France; 3 INSERM, U1018, Center for Research in Epidemiology and Population Health, Department of epidemiology of Occupational and Social Determinants of Health, Villejuif, France; 4 Versailles Saint-Quentin-en-Yvelines University, Villejuif, France; 5 INSERM, UMR912, Economics and Social Sciences Applied to Health and Analysis of Medical Information (SESSTIM), Marseille, France; 6 Aix Marseille University, UMR_S912, IRD, Marseille, France; 7 ORS PACA, Southeastern Health Regional Observatory, Marseille, France; University of Florida, United States of America

## Abstract

**Background:**

Although the various groups of people living with HIV (PLWHIV) considerably differ regarding socioeconomic and behavioral characteristics, their specificities regarding tobacco smoking have been poorly investigated. We aimed to assess patterns of tobacco consumption across the various groups of PLWHIV and to compare them to the general population, accounting for the specific socioeconomic profile of PLWHIV.

**Methods:**

We used data of the ANRS-Vespa2 study, a national representative survey on PLWHIV conducted in France in 2011. Prevalence of past and current tobacco consumption, heavy smoking and strong nicotine dependence were assessed among the various groups of PLWHIV as defined by transmission category, gender and geographic origin, and compared to the French general population using direct standardization and multivariate Poisson regression models, accounting for gender, age, education and geographic origin.

**Results:**

Among the 3,019 participants aged 18–85 years (median time since HIV diagnosis: 12 years), 37.5% were current smokers and 22.1% were past smokers, with marked differences across the various groups of PLWHIV. Compared to the general population, the prevalence of regular smoking was increased among HIV-infected men who have sex with men (MSM) (adjusted prevalence rate ratio (aPRR): 1.19, 95% confidence interval (95% CI): 1.07–1.32), French-native women (aPRR: 1.32, 95% CI: 1.10–1.57), and heterosexual French-native men (although not significantly, aPRR: 1.19, 95% CI: 0.98–1.45). Additionally, HIV-infected MSM were significantly less likely to be ex-smokers (aPRR: 0.73, 95% CI: 0.64–0.82) than the general population and similar trends were observed among heterosexual French-native men (aPRR: 0.89, 95% CI: 0.78–1.02) and women (aPRR: 0.84, 95% CI: 0.70–1.01). HIV-infected sub-Saharan African migrants were less likely to be regular smokers than the general population.

**Conclusions:**

Smoking constitutes a major concern in various groups of PLWHIV in France including MSM and heterosexual French-natives, probably resulting from PLWHIV being less likely to quit smoking than their counterparts in the general population.

## Introduction

With the sustained use of combined antiretroviral therapies (cART), HIV-associated morbidity and mortality have dramatically fallen and the life expectancy of people living with HIV (PLWHIV) has considerably increased over the past two decades [1], [2]. But in the meantime, the burden of non-AIDS related conditions (including cardiovascular diseases and cancers) on PLWHIV’s health has considerably increased [3]–[5]. Among PLWHIV, non-AIDS related co-morbidities occur earlier in life and more frequently than in the general population [6]. Various concurrent pathways have been suggested to explain this increased burden of non-AIDS related conditions among PLWHIV, including the consequences of cART, HIV infection and/or chronic inflammation, as well as a high level of exposure to cardiovascular risk factors [7]–[9]. Non-AIDS related co-morbidities have major consequences on PLWHIV’s mortality, quality of life [10] and social functioning [11]. Thus, they need to be carefully monitored and prevented by the management of modifiable behavioral risk factors.

Tobacco smoking is a major cardiovascular risk factor [12]. Its deleterious consequences on health are particularly marked among PLWHIV [13], [14], among whom it is probably the most widespread and harmful one [15]–[17]. HIV-infected smokers are currently losing more life-years to tobacco than to HIV itself [18].

Previous studies have reported that the prevalence of smoking among PLWHIV in Western countries has been considerably higher than in the general population [13], [16]–[23], ranging from 40% to 70% [5], [13], [16]–[27]. However these previous reports raise various concerns.

First, the rates provided are mainly overall rates, whereas the specificities of the tobacco consumption among the various groups constituting the HIV-infected population have been poorly investigated. These groups of PLWHIV present highly contrasted socioeconomic and behavioral characteristics [28]. Considering that the level of tobacco smoking markedly differs according to these characteristics [29], [30], we hypothesized that the tobacco consumption might be heterogeneous across the various groups of the HIV-infected population.

Secondly, PLWHIV markedly differ from the general population regarding individuals’ socioeconomic characteristics. In Western countries, HIV infection preferentially reaches selected groups of the population with specific socio-demographic and behavioral characteristics that are themselves associated with tobacco consumption. In particular, among HIV-infected people in France the proportions of men who have sex with men (MSM) (39% [31]) and migrants from sub-Saharan Africa (SSA) (24% [31]) are disproportionally high as compared to the French general population (MSM: <1% [32], migrants from SSA: approximately 1% [33]). In addition, history of drug use is disproportionally common among HIV-infected people (11% of past or current intravenous drug users (IDU) [31]) as compared to the French general population (<1% [34]). However, although they are likely to influence the level of tobacco smoking [29], [30], specificities of the HIV-infected population regarding these characteristics have not always been fully accounted for when comparing their tobacco consumption to the general population [13], [16], [17], [19]–[22].

The objectives of this study were 1) to provide detailed information on tobacco smoking among HIV-infected people, at the scale of the whole population of PLWHIV in France in 2011, overall and by group, and 2) to compare the tobacco consumption between the various groups of HIV-infected people and the general population, accounting for the specific socioeconomic profile of PLWHIV.

This will allow improving the knowledge on tobacco smoking across the various groups of PLWHIV and ultimately help better target interventions of smoking prevention or cessation for PLWHIV.

## Methods

### Sources of data

Data on HIV-infected people were obtained from the ANRS-Vespa2 study, a large national representative survey primarily aimed at assessing the various dimensions of socioeconomic conditions and health status of PLWHIV in France in their diversity [35]. The study was conducted between April 2011 and January 2012 in 73 hospital outpatient departments randomly selected among all hospital settings delivering HIV care in metropolitan France. All outpatients aged 18 or older, diagnosed HIV-infected for at least 6 months, and either French-citizens or immigrants living in France for at least 6 months, were eligible. In each participating department, a sample of eligible patients, randomly selected according to the order of their appointment, were invited to participate by their physician.

A total of 5,617 outpatients were randomized. Among them, 378 were not solicited (212 because of major cognitive or health impairment and 166 because of insufficient understanding of the French language) and 2,217 declined to participate. Hence, a total of 3,022 outpatients were included. Participants answered a standardized questionnaire administered face-to-face by a trained interviewer including detailed questions on socioeconomic status, living conditions, health behaviors, health status and healthcare use. Clinical and laboratory information were documented from medical records. Basic characteristics of non-participants were collected anonymously by the physician.

Individual weights were computed to account for unequal probabilities of sampling resulting from the study sampling design and individuals’ annual frequency of hospital appointments. Weights additionally accounted for characteristics associated to non-participation, since participants were more likely to be French citizens, to be unemployed, to have been HIV-infected through homo/bisexual contacts and to have a good immunological status (CD4 cell count>500/mm3) than non-participants.

Information on the general population were obtained from the ‘Baromètre Santé 2010’, the French national health interview survey conducted in 2010 by the French Institute for Health Promotion and Health Education (INPES) [36]. Data were collected by phone among a national representative sample of 27,653 non-institutionalized residents aged 15–85 years.

### Variables of interest

Various indicators of tobacco smoking were computed to assess individuals’ past and current tobacco consumption, based on information collected through the same set of questions in both surveys. Tobacco smoking status was categorized as: *never smokers* (including those who reported having “just tried”); *past smokers* (regardless of past smoking frequency, duration and time since the cessation); and *current smokers*, including *occasional smokers* (<1 cigarette a day) and *regular smokers* (≥1 cigarette a day). Following previously used classifications, among regular smokers, those who reported smoking >10 cigarettes a day were classified as *heavy smokers* [37], and those who either smoked their first cigarette <30 minutes after waking up or smoked >20 cigarettes a day were classified as *strongly nicotine dependent* [25], [38], [39].

Information on socio-demographic characteristics including age, sex, educational attainment, nationality and country of birth was collected in both surveys. Age was categorized into 3 groups (18–39/40–54/55–85 years) consistently with the contrasted profiles and levels of consumption over the lifetime. Level of education, assessed by the highest diploma obtained, was dichotomized as ≤high school degree/>high school degree. Individuals born abroad without the French nationality at birth were classified as migrants.

HIV-infected people were divided into five mutually exclusive categories, which correspond to the most significant groups of PLWHIV in France and markedly differ in terms of socioeconomic conditions and health status [28], [31]: past or current IDU (regardless of sexual orientation and geographic origin); non-IDU MSM (regardless of geographic origin); non-IDU heterosexual French-native individuals (“French-natives”), non-IDU heterosexual migrants from SSA (“SSA migrants”); and non-IDU heterosexual migrants from other regions (“other migrants”).

### Statistical analyses

Analyses were restricted to individuals aged 18–85 years. Considering the marked differences in tobacco smoking figures between men and women [21], [25], [29], [40] all analyses were performed separately by sex.

In a first step, patterns of tobacco smoking were assessed, overall and by group.

Then, these patterns were compared between each group of HIV-infected people and the general population. In this second step, we excluded IDU, who have a very specific behavior towards tobacco smoking regardless of HIV status [41]. We also excluded “other migrants” who constitute a small and heterogeneous group of HIV-infected people. Direct standardization was used to estimate age and education-standardized rates of regular smoking among each group of HIV-infected men and women, considering the general population of same gender as reference. Furthermore, adjusted prevalence rate ratios (aPRR) comparing the prevalence of regular smokers, heavy smokers, strong nicotine dependence and ex-smokers between each group of HIV-infected men and women and the general population of same gender were computed using multivariate Poisson regression models with robust variance [42] adjusted for age, education and an interaction term between these two variables. Moreover, because people originating from SSA are known to have a different smoking profile compared to native individuals in Western countries [43], we considered this group apart from the others and, in addition to comparing tobacco smoking between HIV-infected SSA migrants and the whole general population, we conducted a supplementary analysis restricting the comparison group to the sole SSA migrants of the general population.

All analyses were performed using Stata SE 12.1 (Stata Corporation, College Station, TX) and accounted for data weighting so that the estimates are representative of the whole HIV-infected population followed at hospital in France in 2011.

### Consent of participation and ethical approval

Participants of the ANRS-Vespa2 study signed an informed consent and received a 15€ voucher. The study was approved by the French National Commission for Data Protection and Liberties (CNIL).

## Results

### Study population

Overall, 3,019 HIV-infected participants were aged 18–85 years. They were predominantly men (66.6%) and median time since HIV diagnosis was 12 years (range: 6 months to 29 years). Overall, 13.4% (men: 9.3%; women: 4.1%) were IDU, 36.7% were MSM, 22.4% (men: 10.9%; women: 11.5%) were heterosexual French-natives, 23.7% (men: 7.9%; women: 15.8%) were heterosexual migrants from SSA and 3.8% (men: 1.8%; women: 2.0%) were heterosexual migrants originating from other regions - mainly North Africa (1.2%) and Europe (1.0%). These various groups showed marked differences in terms of age (with a median age ranging from 39 years among heterosexual SSA migrant women to 53 years among heterosexual French-native men) and educational attainment (with a proportion of individuals with more than a high school diploma ranging from 11.1% among heterosexual SSA migrant women to 44.1% among MSM (Table 1).

**Table 1:** Socio-demographic characteristics* of HIV-infected people and the general population in France.

	MEN						WOMEN				
	GENERAL POPULATION	HIV-INFECTED PEOPLE					GENERAL POPULATION	HIV-INFECTED PEOPLE			
	All men	MSM	French-natives	SSA migrants	Other migrants	IDU	All women	French-natives	SSA migrants	Other migrants	IDU
	(N = 11,750)	(N = 1,254)	(N = 313)	(N = 176)	(N = 50)	(N = 281)	(N = 14,947)	(N = 335)	(N = 425)	(N = 61)	(N = 124)
**Age**											
18–40 years	37.7	18.3	9.9	26.2	19.6	5.6	35.2	17.4	51.9	30.8	15.1
40–55 years	28.2	54.8	44.0	55.5	43.9	81.6	27.3	55.8	40.3	44.2	76.5
55–85 years	34.1	26.9	46.1	18.3	36.5	12.8	37.5	26.8	7.8	25.0	8.4
**Education**											
≤high school degree	77.1	55.9	70.8	62.6	82.5	81.9	77.6	72.0	88.9	81.2	86.1
>high school degree	22.9	44.1	29.2	37.4	17.5	18.1	22.4	28.0	11.1	18.8	13.9
**Geographic origin**											
French-natives	91.4	90.7	100.0	0.0	0.0	89.7	91.2	100.0	0.0	0.0	87.3
SSA migrants	1.3	1.6	0.0	100.0	0.0	0.3	1.2	0.0	100.0	0.0	3.6
Other migrants	7.3	7.7	0.0	0.0	100.0	10.0	6.6	0.0	0.0	100.0	9.1

*Weighted percentages.

MSM: Men who have sex with men, SSA: Sub-Saharan African, IDU: Intravenous drug users.

Among the 26,697 respondents of the general population aged 18–85 years, the proportion of men was 47.9%. Median age was 46 years (interquartile range (IQR): 32 to 60 years) among men, and 47 years (IQR: 33 to 62 years) among women. Only 8.6% of men and 7.8% of women were non-French-natives (1.3% and 1.2%, respectively, originating from SSA). Overall, 22.9% of men and 22.4% of women had more than a high school diploma (Table 1).

### Tobacco smoking across the various groups of HIV-infected people

Overall, among HIV-infected people 40.4% were never smokers, 22.1% were past smokers and 37.5% were current smokers (of whom 30.7% were regular smokers and 6.8% were occasional smokers). As shown in Tables 2 and 3, the smoking status varied considerably across the various groups of HIV-infected people, with the highest rates of current smokers among IDU (men: 74.3%; women: 79.9%) and the lowest among heterosexual migrants, particularly those originating from SSA (men: 21.3%; women: 7.9%). Among MSM and heterosexual French-native men and women, 41.8%, 32.8% and 41.2%, respectively, were current smokers. In each group, the large majority of current smokers were regular smokers.

**Table 2:** Prevalence of tobacco smoking among the various groups of HIV-infected men.

	MSM		French-natives		SSA migrants		Other migrants		IDU		p-value**
	%*	[95% CI]	%*	[95% CI]	%*	[95% CI]	%*	[95% CI]	%*	[95% CI]	
*Overall*											
**Smoking status**											
Regular smokers	33.6	[30.4,36.8]	29.1	[23.0,35.2]	13.5	[7.3,19.8]	20.6	[5.4,35.9]	66.7	[59.8,73.5]	<0.001
Occasional smokers	8.2	[6.4,10.0]	3.7	[1.5,5.9]	7.8	[3.4,12.1]	3.8	[–1.3,8.9]	7.6	[3.6,11.7]	
Past smokers	24.6	[21.3,27.9]	38.2	[31.5,45.0]	18.6	[12.2,25.0]	28.2	[13.8,42.7]	21.6	[15.6,27.5]	
Never smokers	33.6	[30.3,36.9]	29.0	[22.3,35.6]	60.1	[51.6,68.6]	47.3	[29.6,65.0]	4.1	[1.5,6.7]	
*Among regular smokers*											
**Heavy smoking**	67.8	[62.7,73.0]	60.3	[49.6,71.0]	38.6	[15.7,61.4]	81.1	[56.9,105.4]	68.8	[61.1,76.5]	0.03
**Strong nicotine dependence**	63.7	[57.9,69.5]	49.0	[37.9,60.1]	29.8	[9.6,50.1]	77.0	[50.3,103.6]	57.2	[48.6,65.9]	0.006
*Among ever smokers*											
**Ex-smokers**	37.0	[32.4,41.5]	53.4	[45.7,61.1]	46.7	[33.3,60.1]	53.6	[30.3,76.8]	22.4	[16.3,28.6]	<0.001

*Weighted percentages.

**χ^2^ test of comparison across the various groups of HIV-infected men.

MSM: Men who have sex with men, SSA: Sub-Saharan African, IDU: Intravenous drug users.

CI: Confidence Interval.

**Table 3:** Prevalence of tobacco smoking among the various groups of HIV-infected women.

	French-natives		SSA migrants		Other migrants		IDU		p-value**
	%*	[95% CI]	%*	[95% CI]	%*	[95% CI]	%*	[95% CI]	
*Overall*									
**Smoking status**									
Regular smokers	32.0	[26.0,38.1]	4.6	[2.0,7.3]	12.1	[2.9,21.3]	71.9	[57.9,85.8]	<0.001
Occasional smokers	9.2	[4.8,13.6]	3.3	[1.5,5.0]	5.3	[–2.0,12.7]	8.0	[–0.2,16.2]	
Past smokers	28.0	[22.1,33.9]	4.0	[1.6,6.5]	15.2	[5.9,24.4]	18.1	[6.8,29.4]	
Never smokers	30.7	[24.7,36.8]	88.1	[84.2,91.9]	67.4	[54.8,80.0]	2.0	[–0.1,4.2]	
*Among regular smokers*									
**Heavy smoking**	55.5	[44.5,66.5]	34.1	[3.0,65.1]	55.8	[15.9,95.7]	64.2	[52.3,76.1]	0.25
**Strong nicotine dependence**	46.7	[35.1,58.3]	36.9	[10.3,63.5]	67.6	[33.0,102.2]	59.6	[47.1,72.2]	0.22
*Among ever smokers*									
**Ex-smokers**	40.5	[32.5,48.5]	33.9	[17.6,50.2]	46.5	[21.6,71.4]	18.5	[7.0,30.0]	0.02

*Weighted percentages.

**χ^2^ test of comparison across the various groups of HIV-infected women.

SSA: Sub-Saharan African, IDU: Intravenous drug users.

CI: Confidence Interval.

Among regular smokers, overall 63.7% were categorized as heavy smokers and 56.9% as strongly nicotine dependent. These rates varied across the groups (Tables 2 and 3), with the lowest rates reported among heterosexual SSA migrant men and women. The highest rates of heavy smoking and strong nicotine dependence were reported among MSM (67.8% and 63.7%), IDU men (68.8% and 57.2%) and women (64.2% and 59.6%), and non-SSA migrant men (81.1% and 77.0%) and women (55.8% and 67.6%).

Among ever smokers, the proportion having quit reached 37.0% overall, varying from 21.2% among IDU (22.4% among men and 18.5% among women) to 37.0% among MSM, 46.8% among heterosexual French-natives (53.4% among men and 40.5% among women), and 50.7% among non-SSA migrants (53.6% among men and 46.5 among women) (Tables 2 and 3).

### Comparison of tobacco smoking between the various groups of HIV-infected people and the general population

After standardization for age and education, the estimated rate of regular smoking reached 33.4% (95% Confidence Interval (CI): 29.7% to 37.2%) among HIV-infected MSM, 33.3% (95% CI: 25.9% to 40.7%) among HIV-infected heterosexual French-native men and 31.1% (95% CI: 24.9% to 37.3%) among HIV-infected French-native women. In the general population, 29.2% of men and 22.9% of women were regular smokers.

As shown in Table 4, HIV-infected MSM were significantly more likely to be regular smokers than men of the general population (aPRR: 1.19, 95% CI: 1.07 to 1.32; p = 0.001) and HIV-infected French-native women were significantly more likely to be regular smokers than women of the general population (aPRR: 1.32, 95% CI: 1.10 to 1.57; p = 0.002). In addition, HIV-infected heterosexual French-native men also had an increased (although not significantly) prevalence of regular smoking (aPRR: 1.19, 95% CI: 0.98 to 1.45]; p = 0.08) compared to men of the general population.

**Table 4:** Adjusted prevalence rate ratios (aPRR) of tobacco smoking for HIV-infected MSM and French-native men and women, as compared to the French general population of same sex.

	MEN			WOMEN		
	aPRR*	[95% CI]	p-value	aPRR*	[95% CI]	p-value
*Overall*						
**Regular smokers**						
General population	1			1		
HIV-infected MSM	1.19	[1.07,1.32]	0.001	-		
HIV-infected French-natives	1.19	[0.98,1.45]	0.08	1.32	[1.10,1.57]	0.002
*Among regular smokers*						
**Heavy smoking**						
General population	1			1		
HIV-infected MSM	1.18	[1.08,1.28]	<0.001	-		
HIV-infected French-natives	1.04	[0.88,1.23]	0.68	1.42	[0.90,2.26]	0.13
**Strong nicotine dependence**						
General population	1			1		
HIV-infected MSM	1.37	[1.24,1.51]	<0.001	-		
HIV-infected French-natives	1.04	[0.83,1.29]	0.75	1.01	[0.78,1.31]	0.96
*Among ever smokers*						
**Ex-smokers**						
General population	1			1		
HIV-infected MSM	0.73	[0.64,0.82]	<0.001	-		
HIV-infected French-natives	0.89	[0.78,1.02]	0.09	0.84	[0.70,1.01]	0.06

*Prevalence rate ratio adjusted for age, education and an interaction term age*education.

MSM: Men who have sex with men.

CI: Confidence Interval.

Among regular smokers, MSM were significantly more likely to be heavy smokers (aPRR: 1.18, 95% CI: 1.08 to 1.28, p<0.001) and strongly nicotine dependent (aPRR: 1.37, 95% CI: 1.24 to 1.51, p<0.001) compared to men of the general population. No difference with the general population in the prevalence of heavy smoking and strong nicotine dependence was found for heterosexual French-native men and women (Table 4).

Among ever smokers, HIV-infected MSM were significantly less likely to be ex-smokers than men of the general population (aPRR: 0.73, 95% CI: 0.64 to 0.82; p<0.001). HIV-infected heterosexual French-native men and women were also less likely (although not significantly) to have quit smoking than the general population of same sex (men: aPRR: 0.89, 95% CI: 0.78 to 1.02; p = 0.09, women: aPRR: 0.84, 95% CI: 0.70 to 1.01; p = 0.06) (Table 4).

Among HIV-infected heterosexual SSA migrants, when considering the whole general population as reference, the estimated standardized rate of regular smoking was 14.6% (95% CI: 5.5% to 23.7%) in men and 6.0% (95% CI: 1.3% to 10.6%) in women, compared to 29.2% in men and 22.9% in women of the general population. As shown in Table 5, HIV-infected heterosexual SSA migrant men (aPRR: 0.45, 95% CI: 0.28 to 0.72; p = 0.001) and women (aPRR: 0.15, 95% CI: 0.08 to 0.26; p<0.001) had a significantly lower prevalence of regular smoking than the whole general population of same sex.

**Table 5:** Adjusted prevalence rate ratios (aPRR) of regular smoking for HIV-infected SSA migrants, as compared to the whole French general population and to SSA migrants of the French general population.

	MEN			WOMEN		
	aPRR*	[95% CI]	p-value	aPRR*	[95% CI]	p-value
***Comparison to the whole French general population***						
General population	1			1		
HIV-infected SSA migrants	0.45	[0.28,0.72]	0.001	0.15	[0.08,0.26]	<0.001
***Comparison to SSA migrants of the French general population***						
General population	1			1		
HIV-infected SSA migrants	0.73	[0.35,1.52]	0.40	0.22	[0.08,0.55]	0.001

*Prevalence rate ratio adjusted for age, education and an interaction term age*education.

SSA: Sub-Saharan African.

CI: Confidence Interval.

When considering the sole SSA migrants of the general population as reference, the standardized rate of regular smoking among HIV-infected heterosexual SSA migrant reached 12.3% (95% CI: 5.7% to 18.8%) in men and 3.8% (95% CI: 1.7% to 5.9%) in women, compared to 16.8% in SSA migrant men and 17.8% in SSA migrant women of the general population. As shown in Table 5, HIV-infected heterosexual SSA migrant women were significantly less likely to be regular smokers than SSA migrant women of the general population (aPRR: 0.22; 95% CI: 0.08 to 0.55; p = 0.001), but the prevalence of regular smoking did not differ between HIV-infected heterosexual SSA migrant men and their counterparts in the general population (aPRR: 0.73; 95% CI: 0.35 to 1.52; p = 0.40).

## Discussion

Our findings indicate that overall, almost four in ten (37.5%) HIV-infected people in France are current smokers, most of them smoking regularly (30.7%). The prevalence of regular smoking varied from 4.6% to 71.9% across the various groups of PLWHIV. HIV-infected MSM and heterosexual French-native men had a 1.19-fold increase in the prevalence of regular smoking as compared to men of the general population, and HIV-infected French-native women had a 1.32-fold increase as compared to women of the general population. HIV-infected MSM had significantly higher prevalence of heavy smoking and strong nicotine dependence and they also were significantly less likely to be ex-smokers, than men of the general population.

Overall, we found elevated prevalence of tobacco smoking in the HIV-infected population, although lower than the 40–70% previously reported in France [16], [21], [22] as well as in other Western countries [5], [17]–[20], [23]–[27]. This is likely to reflect the diversity of the population considered in our study, in contrast with previous studies mostly based on data of prospective cohorts [5], [13], [16], [18], [22]–[24], [27] or of studies restricted to specific settings or populations [13], [24], [25], in which some groups (e.g. people infected through homosexual contacts or drug use) are more likely to be included. Regardless of HIV status, MSM and IDU are known to experiment high prevalence of tobacco smoking [21], [25], [41], [44], thus suggesting that smoking rates reported in those previous studies are likely to overestimate the actual situation at the scale of the whole HIV-infected population. The lower smoking prevalence we reported in 2011 compared to older data may also result from a decrease in tobacco smoking among PLWHIV over the past few years [45], [46] following the global descending trend of tobacco consumption in the general population in the early 2000s [40] and from an increased awareness of smoking-associated risks in the HIV-infected population.

Moreover, our results provide evidence for a marked heterogeneity in tobacco consumption across the various groups of PLWHIV in France, as we hypothesized. We highlighted three contrasted profiles: IDU, who constitute massive tobacco smokers; heterosexual migrants from SSA (and, to a lesser extent, migrants from other regions), who are mostly non-smokers; and MSM and heterosexual French-natives, who have intermediate levels of tobacco consumption compared to IDU and migrants. Our estimates of smoking prevalence and intensity are consistent with those previously reported among HIV-infected IDU [21], [22], [25], [41], MSM [21], [22], [25], and SSA migrants [21]. In addition, we found that HIV-infected IDU and MSM have particularly high levels of heavy smoking and nicotine dependence compared to the other groups. Our results on HIV-infected migrants from other regions are difficult to interpret since their number was limited in the study sample (reflecting their small part –3.8% – overall in the HIV-infected population in France in 2011) and because of their heterogeneity in terms of socio-demographic characteristics, geographic origin and migration history [28].

Data on tobacco consumption of HIV-infected people and the French general population were both collected in 2010–2011 using similar questionnaires, thus allowing appropriate comparisons between the two datasets. To adequately account for the specificities of the HIV-infected population regarding socio-demographic and behavioral characteristics, our analyses comparing HIV-infected people and the French general population were adjusted for age and education and stratified by sex. Additionally, the HIV-infected population was stratified by transmission group and geographic origin and IDU were excluded.

We found that the prevalence of regular smoking, heavy smoking and strong nicotine dependence were significantly higher among HIV-infected MSM compared to men of the general population. Considering the low proportion of MSM among men of the French general population [32], it is likely that our male population of reference was mostly constituted of heterosexual men. Since MSM have been reported to smoke more than heterosexual men [29] our findings of increased prevalence of regular smoking, heavy smoking and strong nicotine dependence among HIV-infected MSM may reflect a different smoking behavior of MSM as compared to heterosexual men rather than a difference between HIV-infected and uninfected men. However, in the general population, the difference in smoking prevalence between MSM and heterosexual men has been suggested to be mediated by socio-demographic differences [44], [47]. In the present study, after accounting for socio-demographic characteristics we did not observe any difference in the standardized rates of regular smoking between HIV-infected MSM and heterosexual French-native men, suggesting that in the HIV-infected population as well the difference in smoking prevalence between MSM and heterosexual men may be mediated by socio-demographic differences. This suggests that the excess tobacco smoking we found among HIV-infected MSM compared to men of the general population is unlikely to be explained by differences in the proportion of MSM between the two populations, but might rather be attributable to differences in term of HIV status.

In addition, our results, by providing evidence of increased smoking rates among HIV-infected heterosexual French-native men and women compared to the general population, reveal that smoking constitutes a major concern in these groups of PLWHIV as well. To our knowledge, this study is the first to specifically report on the tobacco consumption of non-migrant heterosexuals, although they account for a substantial part of the HIV-infected population in European countries – almost a quarter (22.4%) in France in 2011. Our findings suggest that HIV-infected heterosexual French-natives should be paid a specific attention regarding tobacco smoking.

Our findings of lower prevalence of ex-smokers among HIV-infected MSM and heterosexual French-native ever smokers compared to the general population additionally provide strong evidence supporting that the increased frequency of tobacco smoking among these groups of PLWHIV may result from a lower rate of smoking cessation. Low rates of smoking cessation have been previously reported among PLWHIV compared to figures in the general population [13], [18], [23], [25], though to our knowledge this study is the first to formally measure this difference, accounting for differences in individuals’ characteristics. Various factors including HIV disease itself, co-morbidities, adverse living conditions and attitudes of healthcare providers have been reported as potential barriers to smoking cessation among PLWHIV [14], [19], [48].

In contrast, we found a lower rate of regular smokers among HIV-infected SSA migrants compared to the general population in France, a finding consistent with a previous study [21]. This most likely reflects the lower tobacco consumption of SSA migrants compared to non-migrants in the French general population (16.9% versus 25.6% in the Baromètre Santé 2010). However, the difference persisted (although attenuated) in women when restricting the comparison to SSA migrants of the general population, probably mostly resulting from a shorter period since arrival in France (median of 9 years versus 11 years, respectively), and thus a lower level of acculturation to local smoking habits, among HIV-infected SSA migrant women compared to their counterparts in the general population.

The national-representative nature of the ANRS-Vespa2 dataset constitutes a major strength of our study, allowing for the estimation of tobacco smoking figures across the diverse groups of PLWHIV in France and their comparison with the general population. However, some limitations should be acknowledged. First, our results do not apply to HIV-infected individuals not attending hospital for HIV care. Nevertheless, HIV care is essentially provided at hospital in France. It has been estimated that only a minority of PLWHIV in France were exclusively followed for HIV care outside the hospital (7.6% [49]). Moreover, since 2006 the experts have recommended that all PLWHIV have an annual checkup at hospital [50]. Therefore, our estimates are likely to apply to the vast majority of PLWHIV in care in France. Second, although our analyses systematically accounted for socio-demographic differences between PLWHIV and the general population, potential residual confounding cannot be excluded, thus precluding from interpreting our measures of differences in tobacco consumption between both populations in terms of causality. Third, IDU, MSM and migrants from SSA, who constitute a substantial part of PLWHIV, only account for minority, hard-to-reach groups in the French general population. Thus our ability to identify these groups using general population data and to compare them to their HIV-infected counterparts was limited. Further studies using ad hoc data are deserved to formally compare tobacco smoking between these groups of HIV-infected people and their HIV-negative counterparts.

In conclusion, this study provides evidence for a marked heterogeneity in tobacco smoking within the HIV-infected population. Among various groups of HIV-infected people in France including MSM and heterosexual French-natives, regular tobacco smoking is more frequent than in the French general population even after accounting for individuals’ socio-demographic characteristics, and this may result from HIV-infected people being less likely to quit smoking than their counterparts in the general population. As HIV infection has shifted to a chronic condition and the burden of co-morbidities on HIV-infected people’s health has increased, tobacco smoking has emerged as a key issue among people living with HIV. In this context, the information provided in this study constitutes a major contribution to comprehensively inform on tobacco smoking among HIV-infected people and to guide future interventions of smoking prevention in this population. More specifically, our findings highlight the need for a close monitoring of tobacco smoking among the various groups of HIV-infected people and for the identification of the various barriers preventing tobacco cessation in these populations in order to develop adapted supportive interventions.

## Appendix

The ANRS-Vespa2 Study Group includes: France Lert (INSERM UMR-S 1018, France.Lert@inserm.fr) and Bruno Spire (INSERM UMR-S 912/ORS PACA), scientific coordinators; Patrizia Carrieri (INSERM UMR-S 912/ORS PACA), Rosemary Dray-Spira (INSERM UMR-S 1136), Christine Hamelin (INSERM UMR-S 1018), Nicolas Lorente (INSERM UMR-S 912/ORS PACA), Marie Préau (INSERM UMR-S 912/ORS PACA) and Marie Suzan-Monti (INSERM UMR-S 912/ORS PACA); with the collaboration of Marion Mora (INSERM UMR-S 912/ORS PACA).

### List of participating hospitals and investigators

Aix-en-Provence, CH Pays d’Aix (T. Allègre, P. Mours, J.M. Riou, M. Sordage); Angers, CHU Hôtel-Dieu (J.M. Chennebault, P. Fialaire, V. Rabier); Annemasse, CH Alpes-Léman (M. Froidure, D. Huguet, D. Leduc); Avignon, Hôpital Henri Duffaut (G. P ichancourt, A. Wajsbrot); Besançon, Hôpital Saint-Jacques (C. Bourdeaux, A. Foltzer, B. Hoen, L. Hustache-Mathieu); Bobigny, Hôpital Avicenne (S. Abgrall, R. Barruet, O. Bouchaud, A. Chabrol, S. Mattioni, F. Mechai); Bondy, Hôpital Jean Verdier (V. Jeantils); Bordeaux, Hôpital Saint-André (N. Bernard, F. Bonnet, M. Hessamfar, D. Lacoste, D. Malvy, P. Mercié, P. Morlat, F. Paccalin, M.C. Pertusa, T. Pistone, M.C. Receveur, M.A. Vandenhende); Boulogne-Billancourt, Hôpital Ambroise Paré (C. Dupont, A. Freire Maresca, J. Leporrier, E. Rouveix); Caen, Hôpital Clémenceau (S. Dargere, A. de la Blanchardière, A. Martin, V. Noyon, R. Verdon); CH de Chambéry (O. Rogeaux); Clermont-Ferrand, CHU Gabriel Montpied (J. Beytout, F. Gourdon, H. Laurichesse); Colombes, Hôpital Louis-Mourier (F. Meier, E. Mortier, A.M. Simonpoli); Creil, CH Laennec (F. Cordier); Créteil, CHIC (I. Delacroix, V. Garrait, B. Elharrar), Hôpital Henri Mondor (S. Dominguez, A.S. Lascaux, J.D. Lelièvre, Y. Levy, G. Melica); Dijon, Hôpital du Bocage (M. Buisson, L. Piroth, A. Waldner); Eaubonne, Hôpital Simone Veil (N. Gruat, A. Leprêtre); Garches, Hôpital Raymond-Poincaré (P. de Truchis, D. Le Du, J.Cl. Melchior); CH de Gonesse (R. Sehouane, D. Troisvallets); CHU de Grenoble (M. Blanc, I. Boccon-Gibod, A. Bosseray, J.P. Brion, F. Durand, P. Leclercq, F. Marion, P. Pavese); La Rochelle, Hôpital Saint- Louis (E. Brottier-Mancini, L. Faba, M. Roncato-Saberan); La Roche-sur-Yon, CHD Les Oudairies (O. Bollengier-Stragier, J.L. Esnault, S. Leautez-Nainville, P. P erré); CH de Lagny Marne-la-Vallée (E. Froguel, M. Nguessan, P. Simon); Le Chesnay, CH de Versailles (P. Colardelle, J. Doll, C. Godin-Collet, S. Roussin-Bretagne); Le Kremlin-Bicêtre, Hôpital de Bicêtre (J.F. Delfraissy, M. Duracinsky, C. Goujard, D. Peretti, Y. Quertainmont); CH du Mans (J. Marionneau); Lens, CH Dr. Schaffner (E. Aissi, N. Van Grunderbeeck); Limoges, CHU Dupuytren (E. Denes, S. Ducroix-Roubertou, C. Genet, P. Weinbreck); Lyon, Hôpital de la Croix-Rousse (C. Augustin-Normand, A. Boibieux, L. Cotte, T. Ferry, J. Koffi, P. Miailhes, T. Perpoint, D. Peyramond, I. Schlienger); Hôpital Édouard-Herriot (J.M. Brunel, E. Carbonnel, P. Chiarello, J.M. Livrozet, D. Makhloufi); Marseille, Hôpital de la Conception (C. Dhiver, H. Husson, A. Madrid, I. Ravaux, M.L. de Severac, M. Thierry Mieg, C. Tomei), Hôpital Nord (S. Hakoun, J. Moreau, S. Mokhtari, M.J. Soavi), Hôpital Sainte Marguerite (O. Faucher, A. Ménard, M. Orticoni, I. Poizot-Martin, M.J. Soavi); Montpellier, Hôpital Gui de Chauliac (N. Atoui, V. Baillat, V. Faucherre, C. Favier, J.M. Jacquet, V. Le Moing, A. Makinson, R. Mansouri, C. Merle); Montivilliers, Hôpital Jacques Monod (N. Elforzli); Nantes, Hôtel-Dieu (C. Allavena, O. Aubry, M. Besnier, E. Billaud, B. Bonnet, S. Bouchez, D. Boutoille, C. Brunet, N. Feuillebois, M. Lefebvre, P. Morineau-Le Houssine, O. Mounoury, P. Point, F. Raffi, V. Reliquet, J.P. Talarmin); Nice, Hôpital l’Archet (C. Ceppi, E. Cua, P. Dellamonica, F. De Salvador-Guillouet, J. Durant, S. Ferrando, V. Mondain-Miton, I. Perbost, S. Pillet, B. Prouvost-Keller, C. Pradier, P. Pugliese, V. Rahelinirina, P.M. Roger, E. Rosenthal, F. Sanderson); Orléans, Hôpital de La Source (L. Hocqueloux, M. Niang, T. Prazuck), Hôpital Porte Madeleine (P. Arsac, M.F. Barrault-Anstett); Paris, Hôpital Bichat - Claude-Bernard (M. Ahouanto, E. Bouvet, G. Castanedo, C. Charlois-Ou, A. Dia Kotuba, Z. Eid-Antoun, C. Jestin, K. Jidar, V. Joly, M.A. Khuong-Josses, N. Landgraf, R. Landman, S. Lariven, A. Leprêtre, F. L’hériteau, M. Machado, S. Matheron, F. Michard, G. Morau, G. Pahlavan, B.C. Phung, M.H. Prévot, C. Rioux, P. Yéni), Hôpital Cochin-Tarnier (F. Bani-Sadr, A. Calboreanu, E. Chakvetadze, D. Salmon, B. Silbermann), Hôpital européen Georges-Pompidou (D. Batisse, M. Beumont, M. Buisson, P. Castiel, J. Derouineau, M. Eliaszewicz, G. Gonzalez, D. Jayle, M. Karmochkine, P. Kousignian, J. Pavie, I. Pierre, L. Weiss), Hôpital Lariboisière (E. Badsi, M. Bendenoun, J. Cervoni, M. Diemer, A. Durel, A. Rami, P. Sellier), Hôpital Pitié-Salpêtrière (H. Ait-Mohand, N. Amirat, M. Bonmarchand, F. Bourdillon, G. Breton, F. Caby, J.P. Grivois, C. Katlama, M. Kirstetter, L. Paris, F. Pichon, L. Roudière, L. Schneider, M.C. Samba, S. Seang, A. Simon, H. Stitou, R. Tubiana, M.A. Valantin), Hôpital Saint-Antoine (D. Bollens, J. Bottero, E. Bui, P. Campa, L. Fonquernie, S. Fournier, P.M. Girard, A. Goetschel, H.F. Guyon, K. Lacombe, F. Lallemand, B. Lefebvre, J.L. Maynard, M.C. Meyohas, Z. Ouazene, J. Pacanowski, O. Picard, G. Raguin, P. Roussard, M. Tourneur, J. Tredup, N. Valin); Hôpital Saint-Louis (S. Balkan, F. Clavel, N. Colin de Verdière, N. De Castro, V. de Lastours, S. Ferret, S. Gallien, V. Garrait, L. Gérard, J. Goguel, M. Lafaurie, C. Lascoux-Combe, J.M. Molina, E. Oksenhendler, J. Pavie, C. Pintado, D. Ponscarme, W. Rozenbaum, A. Scemla), Hôpital Tenon (P. Bonnard, L. Lassel, M.G. Lebrette, T. Lyavanc, P. Mariot, R. Missonnier, M. Ohayon, G. Pialoux, M.P. Treilhou, J.P. Vincensini); Hôtel-Dieu (J. Gilquin, B. Hadacek, L. Nait-Ighil, T.H. Nguyen, C. Pintado, A. Sobel, J.P. Viard, O. Zak Dit Zbar); Perpignan, Hôpital Saint-Jean (H. Aumaître, A. Eden, M. Ferreyra, F. Lopez, M. Medus, S. Neuville, M. Saada); Pontoise, CH René Dubos (L. Blum); Quimper, Hôpital Laennec (P. Perfezou); Rennes, Hôpital de Pontchaillou (C. Arvieux, J.M. Chapplain, M. Revest, F. Souala, P. Tattevin); Rouen, Hôpital Charles-Nicolle (S. Bord, F. Borsa-Lebas, F. Caron, C. Chapuzet, Y. Debab, I. Gueit, M. Etienne, C. Fartoukh, K. Feltgen, C. Joly, S. Robaday-Voisin, P. Suel); Saint-Denis, CH Delafontaine (M.A. Khuong, J. Krausse, M. Poupard, G. Tran Van); Saint-Étienne, CHU Nord (C. Cazorla, F. Daoud, P. Fascia, A. Frésard, C. Guglielminotti, F. Lucht); Strasbourg, Nouvel hôpital civil (C. Bernard-Henry, C. Cheneau, J.M. Lang, E. de Mautort, M. P artisani, M. Priester, D. Rey); Suresnes, Hôpital Foch (C. Majerholc, D. Zucman); Toulon, CHI Chalucet (A. Assi, A. Lafeuillade), Hôpital Sainte-Anne (J.P. de Jaureguiberry, O. Gisserot); Toulouse, Hôpital de La Grave (C. Aquilina, F. Prevoteau du Clary), Hôpital Purpan (M. Alvarez, M. Chauveau, L. Cuzin, P. Delobel, D. Garipuy, E. Labau, B. Marchou, P. Massip, M. Mularczyk, M. Obadia); Tourcoing, CH Gustave Dron (F. Ajana, C. Allienne, V. Baclet, X. de la Tribonnière, T. Huleux, H. Melliez, A. Meybeck, B. Riff, M. Valette, N. Viget); Tours, CHRU Bretonneau (F. Bastides, L. Bernard, G. Gras, P. Guadagnin); Vandoeuvre-lès-Nancy, CHU Brabois (T. May, C. Rabaud); Vannes, CH Bretagne Atlantique (A. Dos Santos, Y. P oinsignon); Villejuif, Hôpital Paul-Brousse, (O. Derradji, L. Escaut, E. Teicher, D. Vittecoq); CHI de Villeneuve-Saint-Georges, (J. Bantsima, P. Caraux-Paz, O. Patey).
